# Supplementary material for: Ultrafast 3D printing with submicrometer features using electrostatic jet deflection
Source: Nat Commun. 2020 Feb 6;11:753. doi: 10.1038/s41467-020-14557-w (PMC7005155; doi:10.1038/s41467-020-14557-w)
Supplement: Supplementary file 1 — Supplementary Information [file 41467_2020_14557_MOESM1_ESM.pdf]

Supplementary Information

Ultrafast 3D printing with submicrometer features using electrostatic jet deflection

Liashenko et al.

Supplementary Information

Ultrafast 3D printing with submicrometer features using electrostatic jet deflection

Ievgenii Liashenko[1,2], Joan Rosell-Llompart[1,3*] & Andreu Cabot[2,3*]

1 Department of Chemical Engineering, Universitat Rovira i Virgili, Av. dels Països Catalans 26, 43007 Tarragona, Spain.
2 Catalonia Institute for Energy Research - IREC, Sant Adrià de Besòs, Barcelona, 08930, Spain.
3 Catalan Institution for Research and Advanced Studies - ICREA, Pg. Lluís Companys 23, 08010 Barcelona, Spain

*email: joan.rosell@urv.cat; acabot@irec.cat

Contents

1. Supplementary Figure 1. Additional finite element analysis results 3
2. Supplementary Figure 2. Influence of jet viscosity on curvature radius 5
3. Supplementary Figure 3. Long walls combining jet deflection and stage translation 6
4. Supplementary Figure 4. Printing on paper 7
5. Supplementary Table 1. Ink compositions.. 8
6. Supplementary Table 2. Dynamic characteristics of mechanical stage 9
7. Supplementary Table 3. Electrical conductivity of inks 10
8. Supplementary Note 1. Acceleration of jet under electrostatic deflection 11
9. Supplementary Note 2. On the effect of the drop size 12
10. Supplementary Note 3. Charge dissipation times 13

1. Supplementary Figure 1. Additional finite element analysis results

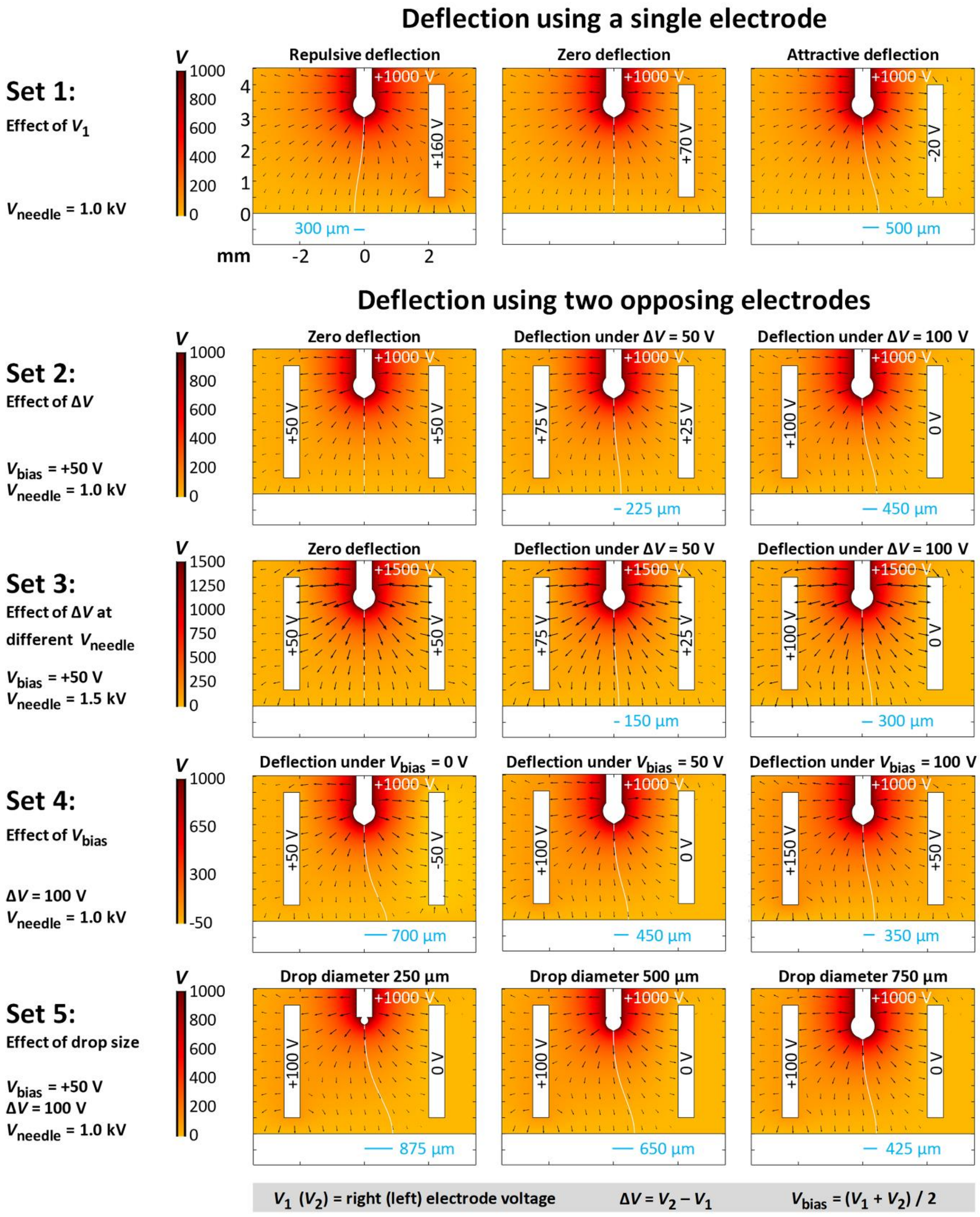

Supplementary Figure 1. Finite element analysis. Simulation of the electric potential and field around the jet in the presence of one and two jet-deflecting electrodes. The nozzle (at the center), the ink drop (hanging at the tip of the nozzle), and the deflecting electrode are shown in white for clarity but are at the specified potentials. The electric field "streamline" (also in white) starting at the tip of Taylor cone (ink drop conical end) represents the theoretical

trajectory of a massless jet. The jet deflections on the collector are written in blue. Five simulation sets were obtained considering different number of deflecting electrodes and voltages. Within each set a different parameter was studied: Set 1 shows jet deflection using one electrode. Voltage bias (+70 V in this case) is required to keep the jet in vertical position. Jet is attracted and repelled from the electrode by applying signal amplitude of ±90 V relative to the bias voltage. The jet deflection distance is greater when the jet is attracted than when it is repelled, 500 µm compared to 300 µm. To correct for this effect and avoid distortion in the printing of predefined objects, our software is designed to dynamically decrease the amplitude when the jet is attracted, and increase when it is repelled. Set 2 shows configuration with 2 electrodes. As deflection signal amplitude is increased to Δ*V*=50 V and Δ*V*=100 V, jet deflection distance is linearly increased. This simulation result is supported by Fig. 2a-b in the manuscript. Set 3 shows the effect of needle voltage by comparing to Set 2. The deflection distance is reduced proportionally to the increase in needle voltage ($\frac{1500\,V}{1000\,V}=\frac{450\ \mu\text{m}}{300\ \mu\text{m}}=1.5$). Set 4 shows how in 2-electrode configuration voltage bias influences the jet deflection. While the amplitude of the deflection voltage is the same (Δ*V*=100 V) for all pictures, the jet is deflected less as the bias increases. Stated differently, increasing the bias has a "focusing effect", which decreases jet deflection. Set 5 shows how drop size changes jet deflection. Simulation shows that a smaller drop results in greater jet deflection. Partially this effect can be attributed to a bigger separation between jet ejection point and substrate. However, the bigger cause of the effect arises from the perturbation of the electric field by the drop itself, i.e. smaller drop perturbs electric field around less (smaller area with red color).

2. Supplementary Figure 2. Influence of jet viscosity on curvature radius

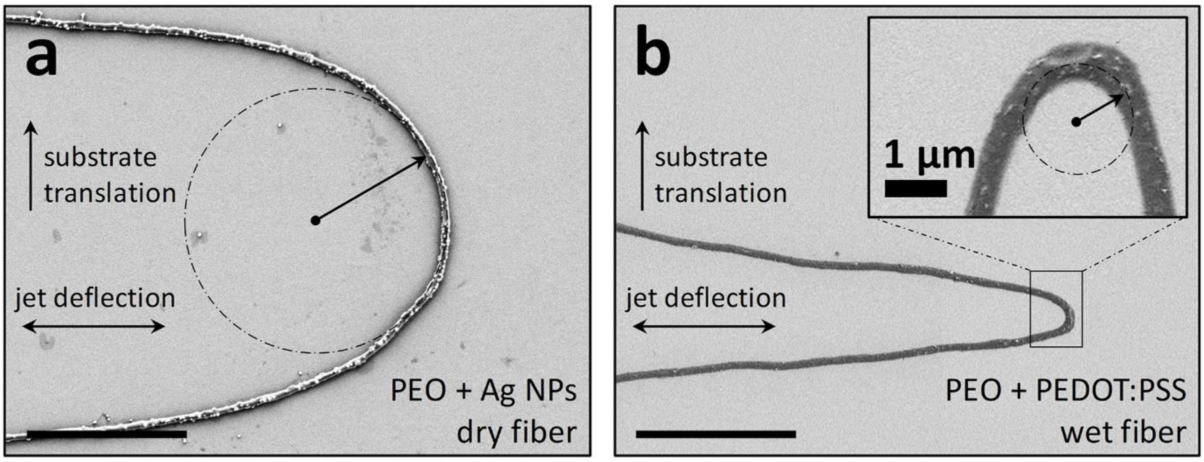

Supplementary Figure 2. **Influence of jet viscosity on curvature radius.** SEM micrographs of lines printed by electrostatically deflecting the jet sideways while the substrate was translated at 5 mm s^{-1}. **a)** Big curvature radius (ca. 10 µm) resulted from a dry-arriving jet produced from an ink containing 10% Ag NPs in 4.5% PEO (300 kDa) in water:ethanol (1:3)) and deflected at 200 Hz using a sawtooth function. **b)** Small curvature radius (ca. 1 µm) resulted from a wet-arriving jet produced from a 8.1% PEDOT:PSS dispersion (3-4%) in 2% PEO (5000 kDa) in water:ethanol (1:1)) and deflected at 100 Hz using a sawtooth function. Scale bars are 10 µm, except for inset.

3. Supplementary Figure 3. Long walls combining jet deflection and stage translation

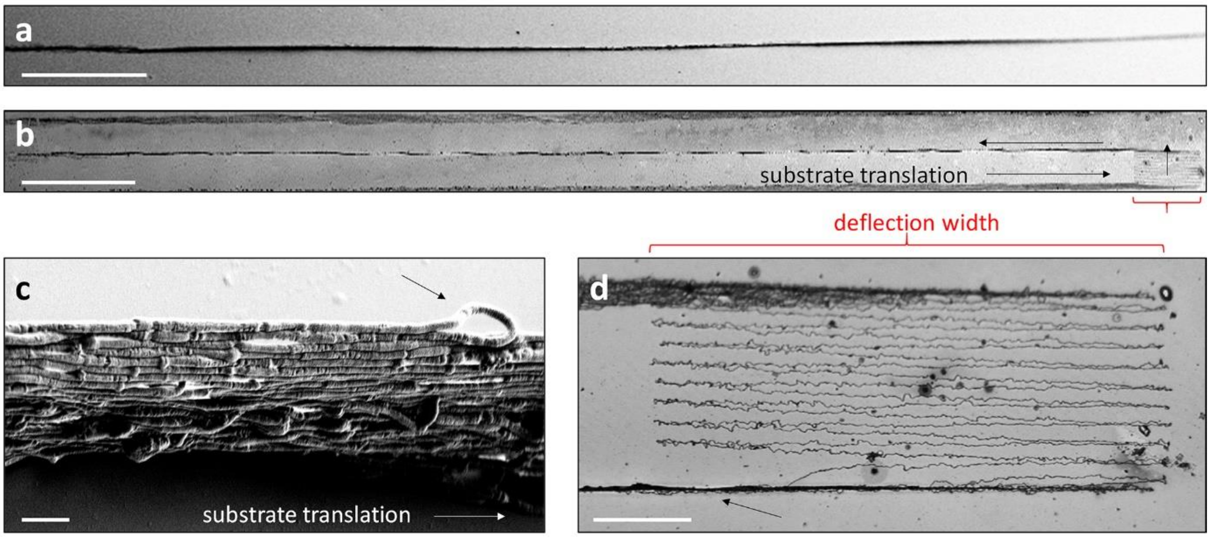

Supplementary Figure 3. Long walls combining jet deflection and stage translation. Confocal and SEM images of PEO walls produced by combining stage translation and jet deflection. **a** SEM image of a long PEO wall printed by jet deflection in the same direction as for substrate translation. Scale bar: 200 µm. **b** Confocal image of a 10 mm long PEO wall (the maximum translation of the XY stage). Scale bar: 1 mm. **c** SEM image showing a magnified view of a PEO wall printed by simultaneous jet deflection and substrate translation. The image displays a fiber loop formed as the fiber was electrostatically deflected back and forth on top of the wall while the substrate was moving towards the right side of the image. Scale bar: 1 µm. **d** Confocal image of the region at the end of a wall where the stage translation direction is changed to print another wall parallel to the first one. At the end of the walls, when the mechanical state moves normal to the jet deflection, the pattern produced by electrostatic jet deflection becomes visible, being much smaller than the wall length defined by the stage translation. Scale bar: 100 µm.

4. Supplementary Figure 4. Printing on paper

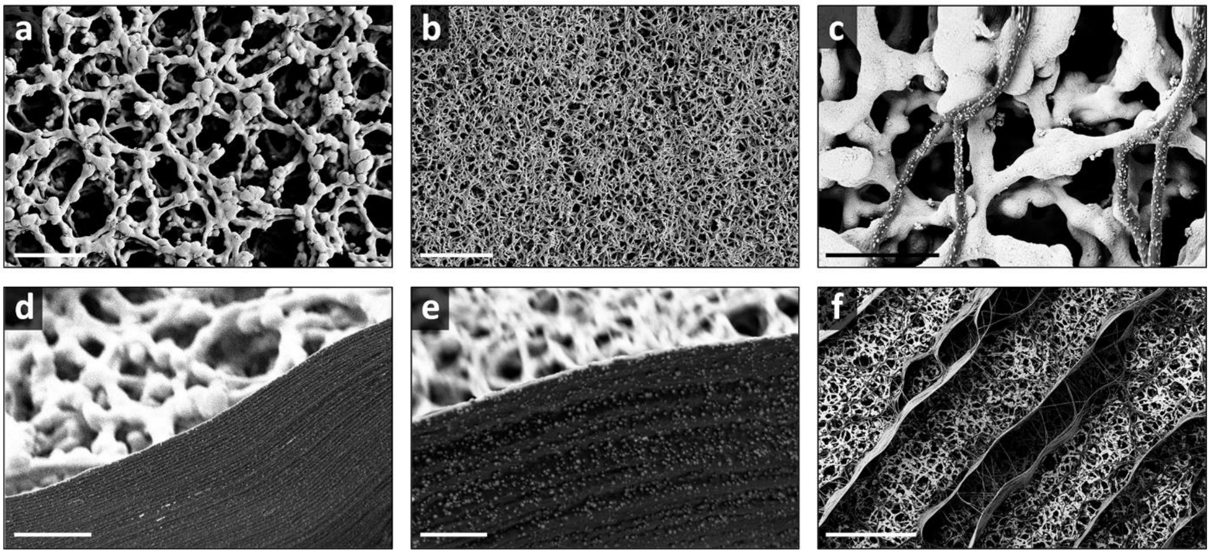

Supplementary Figure 4. Printing on paper. SEM images of porous membranes sputtered with a thin layer of silver prior to printing and of PEO-Ag fibers and walls printed on these porous membranes. **a** Nitrocellulose membrane with polyester backing (Whatman FF 170HP). **b** Nylon filter membrane, pore size 0.22 µm (Sigma-Aldrich #Z290807). Scale bars (**a**-**b**): 10 µm. **c** PEO-Ag fibers printed on the nitrocellulose membrane forming bridges between pores. **d** PEO-Ag wall printed on the nitrocellulose membrane. Scale bars (**c**-**d**): 5 µm. **e** PEO-Ag wall printed on the Nylon filter membrane. Scale bar: 2 µm. **f** Multiple PEO-Ag walls printed on the nitrocellulose membrane. Scale bar: 50 µm. Images (a-c) and (f) are taken from top view, and (d-e) are tilt view.

5. Supplementary Table 1. Ink compositions

Supplementary Table 1. **Ink compositions.** Ink compositions used for the printing of the structures displayed in the manuscript. Percentages are represented in mass (wt.%) relative to the total mass of the ink. Solvent ratios are also represented in mass.

**Ink compositions (wt% and solvent mass ratios)**	**Figures**
10% PEO (300 kDa) in water	5 (b, c, d, e, h); 6 (a)
8% PEO (300 kDa) in water:ethylene glycol (4:1)	4 (b, c, d); 6 (b)
5% PEO (300 kDa) in water:ethanol (1:3)	1 (e)
5% PEO (300 kDa) in water:ethanol (1:1)	2 (a)
2% PEO (5000 kDa) in water:ethanol (1:1)	1 (a)
8.1% PEDOT:PSS dispersion (3-4%) in 2% PEO (5000 kDa) in water:ethanol (1:1).<br>Solid fiber containing 15% PEDOT:PSS and 85% PEO	3 (a, b, c); 6 (f) ; Supplementary Figure 1 (b)
5% Ag NPs in 4.75% PEO (300 kDa) in water:ethanol (1:3)	3 (d, e); 5 (f, g, i, j); 6 (c, d)
10% Ag NPs in 4.5% PEO (300 kDa) in water:ethanol (1:3)	6 (e); Supplementary Figure 1 (a)

6. Supplementary Table 2. Dynamic characteristics of mechanical stage

Supplementary Table 2. **Dynamic characteristics of mechanical stage.** Average speed and acceleration experimentally calculated from a PI miCos PLS-85 mechanical stage as it was moved with maximum speed executing different lineal trajectory travels. The maximum acceleration of the stage was not limited by the software.

Length of line, µm	Number of moves	Total translation time, s	Average speed, mm s^{-1}	Average acceleration, m s^{-2}
5	10000	20	2.50	5.00
10	10000	20	5.00	10.00
20	10000	20	10.00	20.00
50	10000	26	19.23	29.59
100	10000	36.5	27.40	30.02
200	2000	10.2	39.22	30.76
500	2000	16.5	60.61	29.38
1000	2000	27.5	72.73	21.16

7. Supplementary Table 3. Electrical conductivity of inks

Supplementary Table 3. **Electrical conductivity of inks.** Experimental electrical conductivities measured at 25 ºC for inks containing different amounts of PEO, PEO molecular weight, and solvent. Percentages are represented in mass (wt.%) relative to the total mass of the ink. Solvent ratios are also represented in mass.

Ink composition (wt% and solvent mass ratios)	Electrical conductivity (S/cm)
5% PEO (300 kDa) in water:ethanol (1:1)	19.2
5% PEO (1000 kDa) in water:ethanol (1:1)	15.8
2% PEO (1000 kDa) in water:ethanol (1:1)	13.4
10% PEO (300 kDa) in water	99.1
8.1% PEDOT:PSS dispersion (3-4% in water) in 2% PEO (1000 kDa) in water:ethanol (1:1)	19.0

8. Supplementary Note 1. Acceleration of jet under electrostatic deflection

The lateral speed and acceleration reached by the jet under electrostatic deflection were estimated from high-speed video captures of the jet while it was oscillated at 10000 Hz with a sawtooth wave signal. Two jet-deflecting electrodes were positioned on the same deflection axis and 3 mm away from the default jet trajectory (nozzle axis). Nozzle-to-substrate separation was 5 mm. The same voltage amplitudes (max. 1000 V) but opposite signs were applied to the electrodes. The electrified jet was produced from an ink containing 5% PEO (300 kDa) in water:ethanol (1:3) and it was sustained by applying 1500 V between the nozzle and the substrate. A high-speed camera (Photron FASTCAM-1024PCI) was used to capture jet deflection at its maximum rate, 90000 frames per second, and maximum available frame resolution at such high rate (256 pixels wide and 16 pixels high). This captured video strip was obtained 400 μm below the jet-ejection point to observe the jet oscillation. Video captures showed the jet oscillating side to side at 10000 Hz. Through the calibration of microscope and camera, the side-to-side length of this oscillation was determined to be 40 μm.

The average lateral jet speed (v_{LAv}) observed on the video was calculated as a product of the full length travelled by the jet in one oscillation period (40 μm x 2) times the frequency 10000 Hz, giving 0.8 m s^{-1}.

The minimum lateral jet acceleration allowing such an average lateral speed was calculated considering that the jet lateral speed was zero at the extreme side positions, and that the maximum lateral speed (v_{LM}) was reached at the jet's vertical position. Considering a constant lateral jet acceleration (a_{L}), the maximum lateral speed equals twice the average lateral speed, and the constant acceleration of the jet can be computed as: $a_{\mathrm{L}} = \frac{v_{\mathrm{LM}}}{T/4}$, where T is the signal period. This equation results in a lateral jet acceleration of 64,000 m s^{-2}. Considering that the jet is straight at small jet-deflection angles, this calculation was extrapolated to the jet oscillation just above the substrate, 5 mm below the jet-ejection point. In this case, the average jet speed and the minimum required jet acceleration were 12.5 m s^{-1} and 800,000 m s^{-2}, respectively. Considering non-constant accelerations, higher lateral acceleration values would be obtained. Furthermore, much higher accelerations are potentially possible by applying larger voltages to the electrodes, or by positioning them closer to the jet, etc.

Thus, from the conservative calculation presented here we conclude that jet lateral acceleration caused by electrostatic deflection could be significantly higher than 500,000 m s^{-2}, the value provided in the manuscript for the sake of comparison to the accelerations typically reached by mechanical stages.

9. Supplementary Note 2. On the effect of the drop size

The drop size affects printing in different, direct and indirect ways. We experimentally found that smaller drops required smaller voltage applied to the needle to initiate the jet. This experimental observation can be rationalized considering that, at equal voltage applied, a higher electric field and a higher field gradient are generated on the surface and at the surrounding of smaller drops. Results of finite element analysis of the electric field with different drop sizes support this view (Supplementary Figure 1).

On the other hand, smaller drops resulted in slower solvent evaporation rates, which resulted at the site of ejection of the EHD jet in a lower polymer concentration than larger drops. The main consequence of this is that smaller drops lead to a more easily deformable (less viscous) ink at the jet, thus a faster EHD jet (for the same electric field strength, despite a lower voltage is used). The drop size thus also influences the viscoelastic properties of the jet on its arrival to the substrate. Larger drops result in a more viscous jet arriving to the substrate and thus produce a more porous final 3D solid structure since different layers do not fuse together. The results obtained from the deposition of jets with variable dryness is displayed in Fig. 6 and Supplementary Figure 2. Additionally, solute enrichment at the interface of the drop can cause changes in the surface tension. A reduction in surface tension will result in a reduction in the required voltage needed to sustain the jet. In this case, the same deflection will be obtained with a lower deflection signal amplitude (ΔV).

Additionally, the effect of drop size on the electrical field causing the jet to deflect was further investigated via finite element analysis (Supplementary Figure 1). Finite element analysis shows that large drops substantially decrease the jet deflection distance. This result is explained considering two dependences: i) For small droplets, the vertical electric field strength decays faster as the jet moves away from the drop. As the vertical component of the electric field is weaker, the jet-deflecting field (horizontal field component) becomes relatively stronger, thus resulting in larger horizontal deflection of the jet. ii) While the nozzle elevation is kept constant, the jet ejection point is lower for larger drops. Thus, even at equal deflection angle, the jet contact point at the substrate will be moved a shorter horizontal distance.

10. Supplementary Note 3. Charge dissipation times

The charge relaxation time (τ) of a material can be computed from its dielectric permittivity (ε) and electrical conductivity (σ)[1]:

$$\tau = \frac{\varepsilon}{\sigma}$$

Considering the electrical conductivities experimentally measured from the inks and the dielectric constant of water, the relaxation time of the ink was 3×10^{-7} s.

On the other hand, considering the electrical conductivity of PEO on the order of 10^{-9} S/cm (Ahmed, H. T. & Abdullah, O. G. Preparation and composition optimization of PEO: MC polymer blend films to enhance electrical conductivity. Polymers (Basel). 11, 1–18, 2019) and a dielectric constant on the order of 10,[2] we obtained a PEO relaxation time on the order of 10^{-3} s.

When printing PEO patterns, charge dissipates to the high electrical conductivity silicon through wet PEO, which has a higher electrical conductivity than dry PEO, thus the effective relaxation times were lower than 10^{-3} s, in the range from 10^{-7} s to 10^{-3} s.

Supplementary References

1. Melcher, J. R. & Taylor, G. I. Electrohydrodynamics: A Review of the Role of Interfacial Shear Stresses. *Annu. Rev. Fluid Mech.* **1**, 111–146 (1969).
2. Kliem, H., Schröder, K., & Bauhofer, W. High dielectric permittivity of polyethylene oxide in humid atmospheres. P*roceedings of Conference on Electrical Insulation and Dielectric Phenomena - CEIDP '96*, 12-15 (1996).
