# Supplementary material for: Ultrafast 3D printing with submicrometer features using electrostatic jet deflection
Source: Nat Commun. 2020 Feb 6;11:753. doi: 10.1038/s41467-020-14557-w (PMC7005155; doi:10.1038/s41467-020-14557-w)
Supplement: Supplementary file 3 — Description of Additional Supplementary Files [file 41467_2020_14557_MOESM3_ESM.pdf]

Description of Additional Supplementary Files

File Name: Supplementary Movie 1
Description: High-speed video of the jet being deflected in 1D with a frequency of 10, 50, and 100 Hz. The two jet-deflecting electrodes (not shown) were positioned on the left and right sides of the image plane. The video shows the nozzle, the Taylor cone at the end of the ink drop and the thin jet expelled. The trajectory of this jet and thus its point of arrival to the substrate (not shown, positioned 2 mm below the bottom of the image) were modulated by the voltage applied to the jet-deflecting electrodes. The video was recorded with a high-speed camera (Photron FASTCAM-1024PCI) mounted on a microscope and operated at 1000 fps and 1/3000 s shutter speed, under dark-field setting. The video playback speed is 33 times slower than the real video speed.

File Name: Supplementary Movie 2
Description: High-speed video displaying the printing of a cylindrical structure at a frequency of 200 Hz (200 layers per second). Cylindrical structures comprising 100 layers were printed in 0.5 second intervals while the substrate was not moved. Between consecutive printing events the substrate was rapidly moved by the mechanical stage (PI miCos linear stages PLS-85) at 20 mm s-1. The walls of the resulting structures were vertical during printing, as seen on the video. However, subsequent examination of the same structures on SEM revealed the walls to be a bit tilted (due to drying and shrinking). The PEO-based jet had a diameter of ca. 200 nm and it is invisible on this video. The video was recorded with a high-speed camera (Photron FASTCAM-1024PCI) mounted on microscope at 1000 fps and 1/3000 s shutter speed, under dark-field illumination. Video playback speed is 20 times slower than for the real printing process.

File Name: Supplementary Movie 3
Description: Compilation of results from finite element analysis of the change of the electric potential and field around the jet in the presence of two jet-deflecting electrodes when the voltage at these electrodes is continuously changed. The electric field "streamline" plotted in white and starting at the tip of Taylor cone represents the theoretical trajectory of a massless jet.
